# Cuproptosis-related gene signature correlates with the tumor immune features and predicts the prognosis of early-stage lung adenocarcinoma patients

**DOI:** 10.3389/fgene.2022.977156

**Published:** 2022-09-14

**Authors:** Yu Tang, Qifan Li, Daoqi Zhang, Zijian Ma, Jian Yang, Yuan Cui, Aiping Zhang

**Affiliations:** ^1^ Department of Thoracic and Cardiovascular Surgery, Nanjing First Hospital, Nanjing Medical University, Nanjing, China; ^2^ Department of Thoracic Surgery, The First Affiliated Hospital of Soochow University, Suzhou, China; ^3^ Institute of Thoracic Surgery, The First Affiliated Hospital of Soochow University, Suzhou, China; ^4^ Department of Internal Medicine Teaching and Research Section, Xuancheng Vocational and Technical College, Xuancheng, China

**Keywords:** early-stage lung adenocarcinoma (es-LUAD), signature, TME, risk score, cuproptosis

## Abstract

**Background:** Although a majority of early-stage lung adenocarcinoma (es-LUAD) patients have a favorable prognosis, there are still some cases with a risk of recurrence and metastasis. Cuproptosis is a new form of death that differs from other programmed cell death. However, no study has been reported for setting a prognostic model of es-LUAD using cuproptosis pattern-related genes.

**Methods:** Using multiple R packages, the data from the GEO database was processed, and es-LUAD patients was classified into two patterns based on cuproptosis-related genes. Key differentially expressed genes (DEGs) in the two patterns were screened to construct a prognostic signature to assess differences in biological processes and immunotherapy responses in es-LUAD. Tumor microenvironment (TME) in es-LUAD was analyzed using algorithms such as TIMER and ssGSEA. Then, a more accurate nomogram was constructed by combining risk scores with clinical factors.

**Results:** Functional enrichment analysis revealed that DEGs in two patterns were correlated with organelle fission, nuclear division, chromosome segregation, and cycle-related pathways. Univariate Cox regression and Lasso-Cox regression analyses identified six prognostic genes: ASPM, CCNB2, CDC45, CHEK1, NCAPG, and SPAG5. Based on the constructed model, we found that the high-risk group patients had higher expression of immune checkpoints (CTLA4, LAG3, PD-L1, TIGIT and TIM3), and a lower abundance of immune cells. Lastly, the nomogram was highly accurate in predicting the 1-, 3-, and 5-year survival status of patients with es-LUAD based on risk scores and clinical factors.

**Conclusion:** The cuproptosis pattern-related signature can serve as a potential marker for clinical decision-making. It has huge potential in the future to guide the frequency of follow-up and adjuvant therapy for es-LUAD patients.

## Introduction

Lung cancer has the highest mortality rate and the second incidence of all cancer worldwide (Alexander et al., 2020; Sung et al., 2021). Non-small cell lung cancer (NSCLC) comprises approximately 85% of lung cancers, and lung adenocarcinoma (LUAD) is the most common histological subtype of NSCLC (Anusewicz et al., 2020). With the development of targeted therapy and immunotherapy, the prognosis of lung cancer has been greatly improved. Through the promotion of low-dose chest computerized tomography (CT) scan, the diagnosis rate of early-stage lung cancer (es-LUAD) is getting higher (National Lung Screening Trial Research et al., 2013). Early diagnosis can improve the prognosis of lung cancer, with 5-year relative survival increasing from 6% for distant-stage disease to 33% for regional stage and 60% for localized-stage disease (Siegel et al., 2022). Low-dose chest CT scan for lung cancer has become a standard of care in the United States (Mazzone et al., 2021). However, not all the early-diagnose LUAD can be cured. A reliable prognostic signature for es-LUAD patients is needed to improve treatment strategies.

Programmed cell death (PCD) is necessary for the process of eliminating the loss of the damaged infected or senescent cells. The mechanism of PCD included Apoptosis (Kerr et al., 1972), Necroptosis (Sun et al., 2012), Autophagy (Bergmann, 2007), Ferroptosis (Dixon et al., 2012), Proptosis (Shi et al., 2015), and Necrosis (Vanlangenakker et al., 2012). Recently, cuproptosis as a potential factor for cancer disease, can lead to a new form of programmed copper-induced cell death by the mitochondrial tricarboxylic acid (TCA) cycle (Tsvetkov et al., 2022). Too little or too much copper is toxic to cells. The accumulation of copper in the mitochondria results in aggregated lipoylated proteins, including dihydrolipoamide S-acetyl transferase (DLAT) (Tsvetkov et al., 2022). However, whether cuproptosis-related genes could become an important biological marker for predicting early lung cancer needs to be further explored.

Immunotherapy is a promising treatment for es-LUAD, which kills tumor cells by stimulating specific immune responses to diminish tumor immune escape. Clinical studies showed that immunotherapy has a good effect on advanced NSCLC (Garon et al., 2019). Moreover, early-stage surgically respectable LUAD may benefit from Immunotherapy (Linehan and Forde, 2020). Bioinformatics analysis of immune checkpoint expression levels and immune cell infiltration is warranted to help predict immunotherapy efficacy and facilitate precision treatment of es-LUAD.

Considering the findings, we performed a study to find out the relationship between cuproptosis-related genes and es-LUAD. In addition, a clinical prediction model based on the cuproptosis patterns was developed to investigate the correlation of risk scores with prognosis and TME.

## Methods

### Data source

All data of es-LUAD, including RNA-seq data and corresponding clinical date (GSE31210, GSE50081, GSE72094), were retrieved from the Gene Expression Omnibus (GEO) database from the NCBI (https://www.ncbi.nlm.nih.gov/gds/). The expression profiles in the datasets were normalized. After removing the samples with missing survival data, 353 samples with pathological stage I and II in GSE31210 and GSE50081 datasets were obtained. Subsequently, the two datasets were merged by the R package “inSilicoMerging” (Taminau et al., 2012). The ComBat method in the “sva” package was used to eliminate batch effects of the merged dataset (Johnson et al., 2007). In addition, the LUAD patients were extracted from the GSE72094 database with pathological stage I and II as an external validation set to assess the predictive accuracy of the signature. The details of the databases are placed in Supplementary Table S1.

### Consensus clustering

By using the R package “ConsensusClusterPlus” (Wilkerson and Hayes, 2010), unsupervised consensus clustering was applied to the expression profile of the 10 cuproptosis-related genes in the training dataset. Agglomerative km clustering was used with a euclidean distances and resampling 80% of the samples for 1000 repetitions. The stability between different subtypes is best when parameter K = 2. The detailed information was showed in Supplementary Figure S1.

### Functional enrichment analysis

The R package “limma” was used to screen cuproptosis-related differentially expressed genes (CR-DEGs) between two patterns (cut-off criteria: |Fold Change|>1.5 and FDR<0.05) (Ritchie et al., 2015). GO (Gene Ontology) and KEGG (Kyoto Encyclopedia of Genes and Genomes) analyses were performed using R package “clusterProfiler” (FDR<0.05) (Kanehisa et al., 2021). Gene Set Enrichment Analysis (GSEA) was achieved by GSEA software (v 3.0), and different functional phenotypes between two patterns were detected based on KEGG gene sets (Subramanian et al., 2005). R package “GSVA” was used to investigate the biological processes related to high- and low-risk groups. Hallmark gene sets were downloaded from MsigDB (http://www.gsea-msigdb.org/gsea/msigdb/) (Hanzelmann et al., 2013).

### Construction of risk model and nomogram

Protein-Protein Interaction (PPI) network was performed with the STRING database. Cytoscape software was used to screen hub CR-DEGs. On the basis of hub DEGs, the prognostic model was constructed through univariate Cox regression and Lasso-cox regression analyses. The model formula is Risk score = coef1*exp (gene1) +coef2*exp (gene2) +……+coefi*exp (genei). Combining risk scores with clinical factors, we constructed a nomogram utilizing R package “rms.” The 1-/3-/5‐year overall survival (OS) probabilities were estimated by time‐dependent ROC curves and the Concordance index (C‐index) was used to evaluate discriminative ability.

### Statistical analysis

Based on the R package “survminer” and “survival,” Kaplan-Meier (KM) survival analysis was performed to assess prognostic differences. In addition, the correlation between immune cell infiltration and risk score was investigated by the TIMER algorithm and single sample GSEA (ssGSEA) algorithm. Lasso-Cox regression analysis was conducted using the R package “glmnet” (Tibshirani, 1997; Wang et al., 2019). A predictive Nomogram was further constructed based on Cox regression analysis. Time‐dependent ROC curves were drawn by R package “timeROC.” The chi-square test was utilized to evaluate differences between categorical variables, and the *t*-test was applied to continuous variables, in which a nominal *p*-value < 0.05 was significant, and *p* ≥ 0.05 was equal to no significance (NS).

## Result

### Identification of es-LUAD patterns by cuproptosis-related genes

**FIGURE 1:**
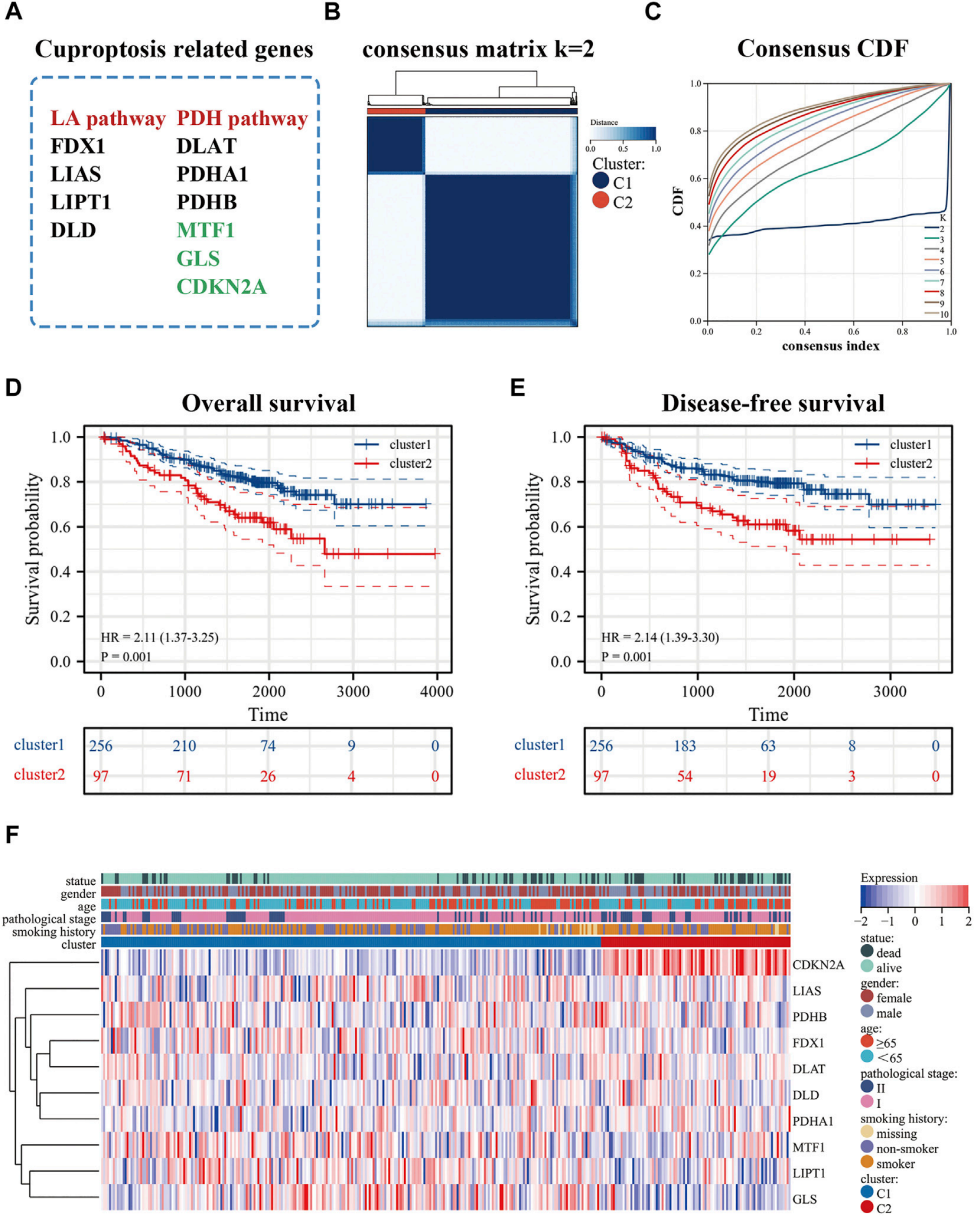
Identification of cuproptosis-related patterns. **(A)** cuproptosis-related genes; **(B,C)** Consensus clustering analysis by the cuproptosis-related genes (k = 2); **(D,E)** KM analysis was used to plots OS and DFS curves of es-LUAD patitents (blue represented cluster1 pattern while red represented cluster2 pattern); **(F)** The heat map showed the expression and clinicopathological characteristics of the cuproptosis-related genes in two clusters. KM: Kaplan-Meier; OS: Overall survival; DFS: Disease-free survival; es-LUAD: early-stage lung adenocarcinoma.

The flowchart was shown in Supplementary Figure S2. After normalization, GSE31210 and GSE50081 were combined as the training set and the batch effects were eliminated. These processes were a prerequisite for subsequent bioinformatics analysis. cuproptosis-related genes derived from recent studies (Kahlson and Dixon, 2022; Tsvetkov et al., 2022) were associated with the LA pathway (FDX1, LIAS, LIPT1, DLD), and the PDH pathway (DLAT, PDHA1, PDHB, MTF1, GLS, CDKN2A) (Figure 1A). In addition, we used an empirical cumulative distribution function (CDF) to determine the optimal number of clusters. Briefly, to identify tumors with common genetic signatures, the training set was subjected to a consensus clustering algorithm (input k = 2–10), based on the cuproptosis-related gene expression profiles (Supplementary Figure S2). When the parameter K = 2, the stability between the two patterns was the best. The cluster1 pattern contained 256 patients, and the cluster2 pattern contained 97 patients (Figures 1B,C). Remarkably, the predicted prognosis was analyzed by the Kaplan-Meier (KM) survival curve. The cluster2 group had notably shorter overall survival compared with the cluster1 group (hazard ratio (HR): 2.11, CI: 1.37–3.25), as was disease-free survival (HR: 2.14, CI: 1.39–3.30). All cohorts showed a significant *p*-value (*p* < 0.001) (Figures 1D,E). In addition, the heat map displayed the expression of cuproptosis-related genes in the two patterns and the demographic information of patients (Figure 1F). Importantly, the results displayed that cuproptosis genes may be essential for es-LUAD patients, and the clinical value was related to overall and disease-free survival.

### Functional enrichment analysis of CR-DEGs

**FIGURE 2:**
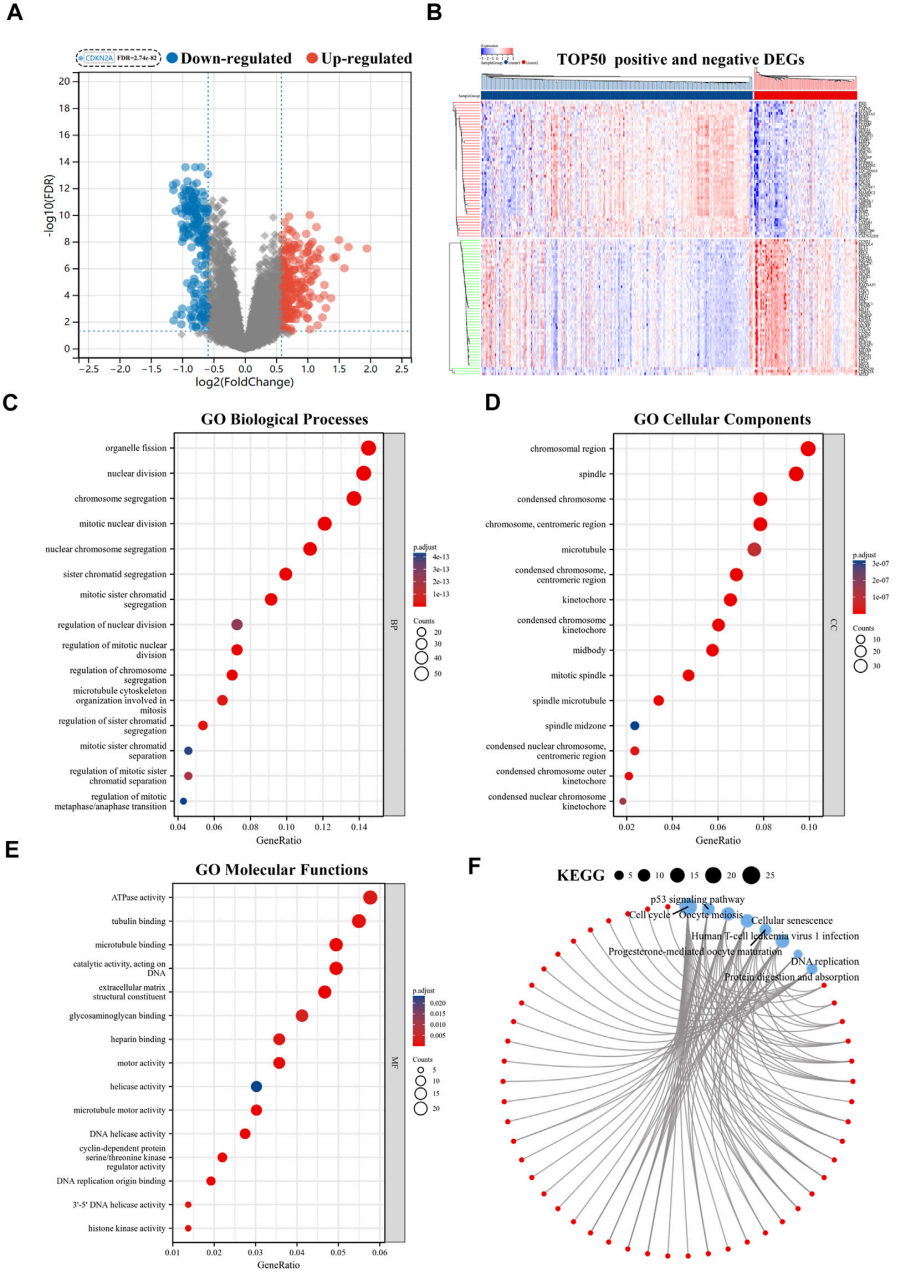
Functional enrichment analyses of CR-DEGs. **(A)** Volcano plot for the CR-DEGs with statistical significance (the green dots represented down-regulated genes, and the red dots represented up-regulated genes); **(B)** The heat map showed the expression of top 50 up-and down-regulated CR-DEGs; **(C–E)** GO analysis of CR-DEGs (|Fold Change|>1.5 and FDR<0.05); **(F)** KEGG analysis of CR-DEGs (|Fold Change|>1.5 and FDR<0.05). CR-DEGs: cuproptosis-related differentially expressed genes; GO: Gene Ontology; KEGG: Kyoto Encyclopedia of Genes and Genomes.

**FIGURE 3:**
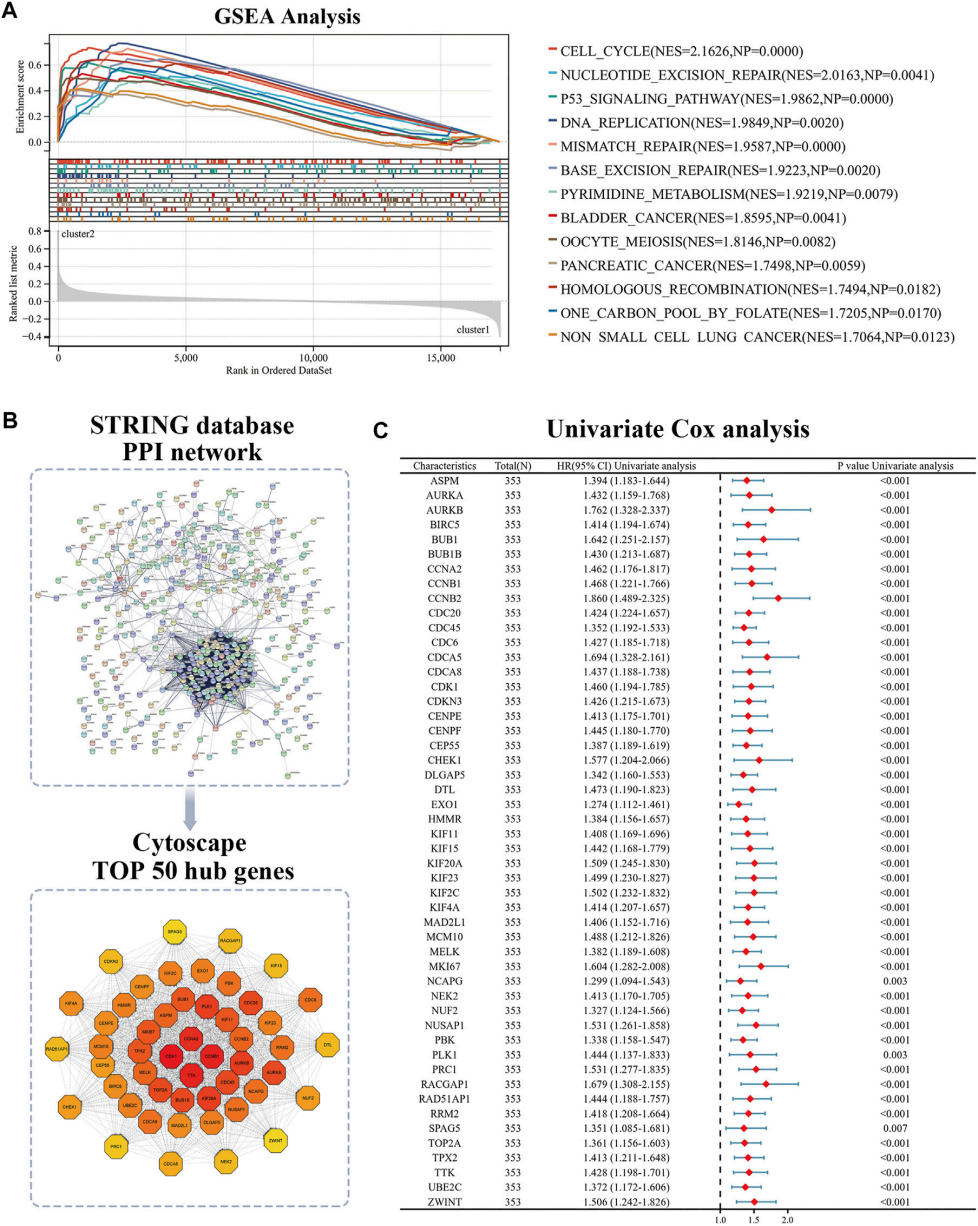
Identification hub genes in PPI network. **(A)** GSEA analysis of two patterns; **(B)** The STRING database and Cytoscape software was used to obtain hub CR-DEGs; **(C)** The hub 50-DEGs analyzed by Univariate Cox regression. PPI: Protein-Protein Interaction; GSEA: Gene Set Enrichment Analysis.

The volcano plot identified 415 CR-DEGs in cluster 1 and cluster 2. The heat map contained the top 50 up- and down-regulated genes, with the columns representing sample groups, and rows representing genes (Figures 2A,B). Functional enrichment analysis was applied to estimate the functions of the CR-DEGs in LUAD. The results of GO enrichment analysis showed that most DEGs were correlated with organelle fission, nuclear division, and chromosome segregation, which belong to the category of biological processes. The most abundant terms in the cellular composition categories were chromosomal region, spindle, and condensed chromosome. For the molecular functions, the strongest enrichment terms were ATPase activity, tubulin binding, and microtubule-binding (Figures 2C–E). KEGG results showed that CR-DEGs were mainly enriched in cycle-related pathways, for example, cell cycle, P53 signaling pathway, and Oocyte meiosis (Figure 2F). More detailed information is shown in Supplementary Table S2. Furthermore, GSEA was implemented to assess the functional differences between the two patterns. The results showed that cluster2 pattern has the actively enrichment of cell cycle pathways, such as cell cycle (NES = 2.16), DNA replication (NES = 1.98), Mismatch repair (NES = 1.96), and Oocyte meiosis (NES = 1.81) in cluster2 group (Figure 3A; Supplementary Table S3)

### Protein-protein interaction networks (PPIs) of CR-DEGs

To explore the interrelationships among DEGs and to find the critical DEGs for subsequent analysis, we used the STRING database and Cytoscape software for visualization. The STRING database is a powerful tool which was used to efficiently perform protein interaction analysis and build PPI network. All hub genes in the PPI network were obtained by the Cytoscape software, and the CytoHubba plugin was used to calculate each Degree score (Figure 3B). Finally, the TOP50 hub DEGs were screened and univariate Cox regression was conducted to define the candidate prognosis-correlated CR-DEGs (Figure 3C).

### Development of prognostic signature

**FIGURE 4:**
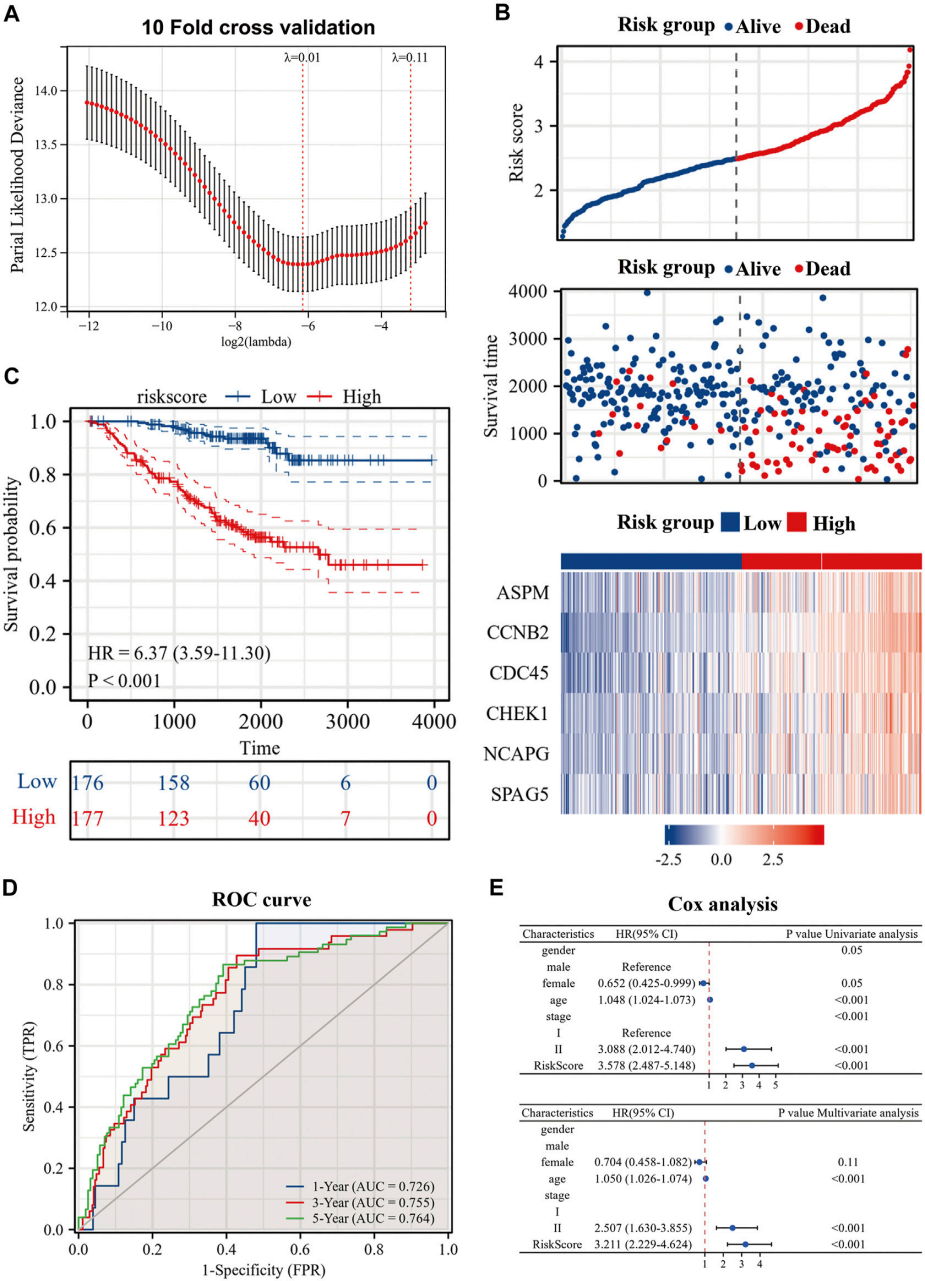
Construction of risk model by six genes. **(A)** Construction of prognostic signature by Lasso regression and 10-fold cross validation; **(B)** Risk factor diagram of es-LUAD patients; **(C)** KM prognostic curve of training group; **(D)** ROC curve of training group; **(E)** Univariate Cox analysis and Multivariate Cox analysis of risk score. Lasso: Least absolute shrinkage and selection operator; KM: Kaplan-Meier; ROC: receiver operating characteristic.

To further explore the prognostic guidance of the top 50 CR-DEGs for patients with es-LUAD, the Lasso-Cox regression was conducted (10-fold cross-validation). Ultimately, we identified six prognostic genes: ASPM, CCNB2, CDC45, CHEK1, NCAPG, and SPAG5 (Figure 4A). These six prognostic genes were used to construct the risk model, and Figure 4B displayed the risk score and survival status of LUAD patients. Moreover, the heat map showed the expression of six prognostic genes between the high- and low-risk groups. According to the survival probability analysis, the survival probability of the high-risk group was markedly lower than that of the low-risk group (HR = 6.37, CI: 3.59–11.30) (Figure 4C). Then, time-dependent ROC curves were used to verify the diagnostic efficiency of the signatures. The results showed that the predicted AUC values of 1-, 3-, and 5-year survival in pathological stage I and II LUAD patients were 0.726, 0.755, and 0.764, respectively (Figure 4D). Univariate and multivariate Cox analyses were applied to further investigate the prognostic correlation of risk scores. Results showed that risk score was an independent prognostic factor in es-LUAD patients (Figure 4E).

### Risk scores correlated with clinicopathological factors and reveal differences in biological functions

**FIGURE 5:**
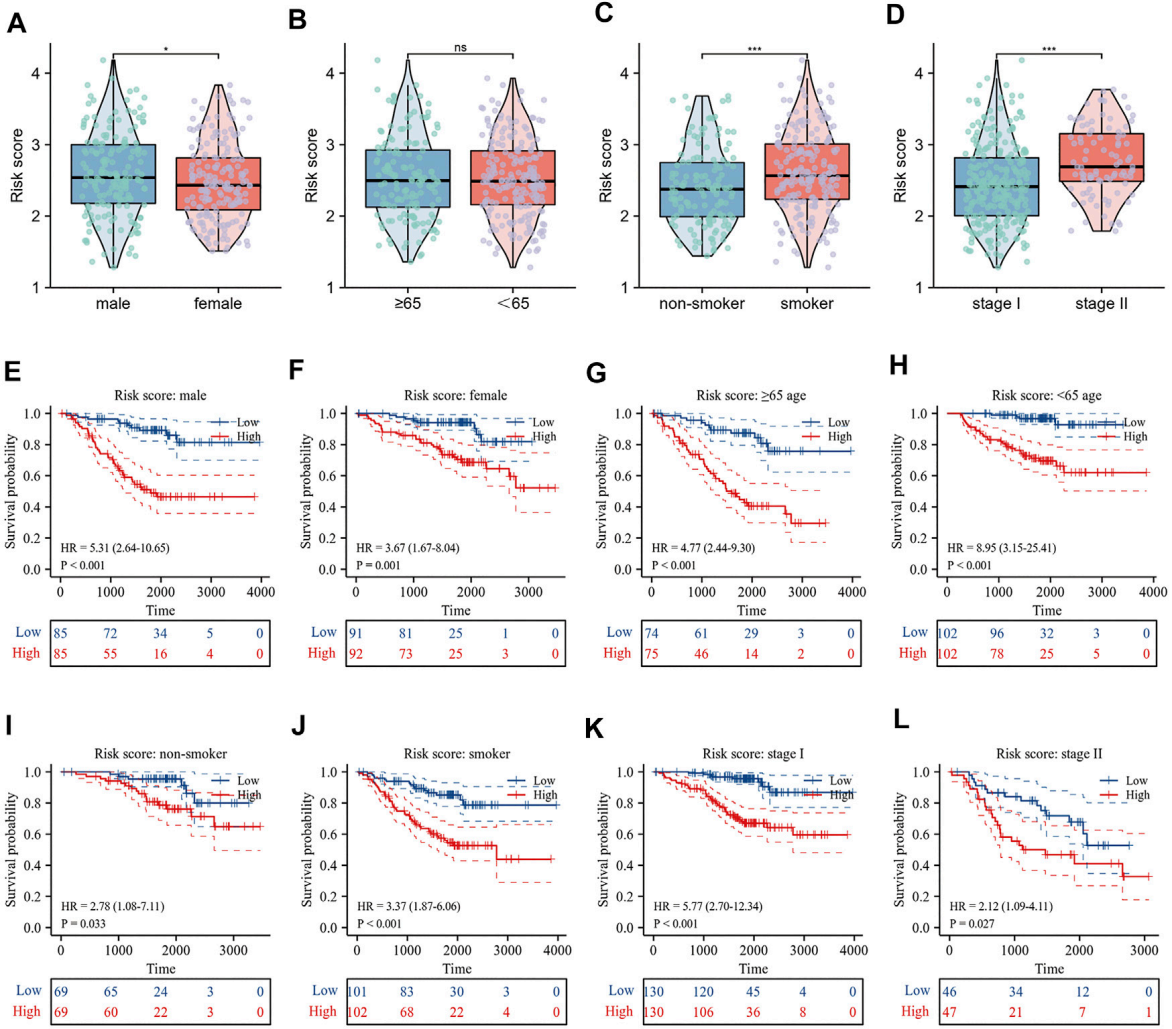
Relationship between risk score and clinicopathological characteristics. **(A–D)** Differences in risk score by gender, age, smoking history and pathological stage; **(E–L)** Prognostic significance of risk score in different clinical factors.

**FIGURE 6:**
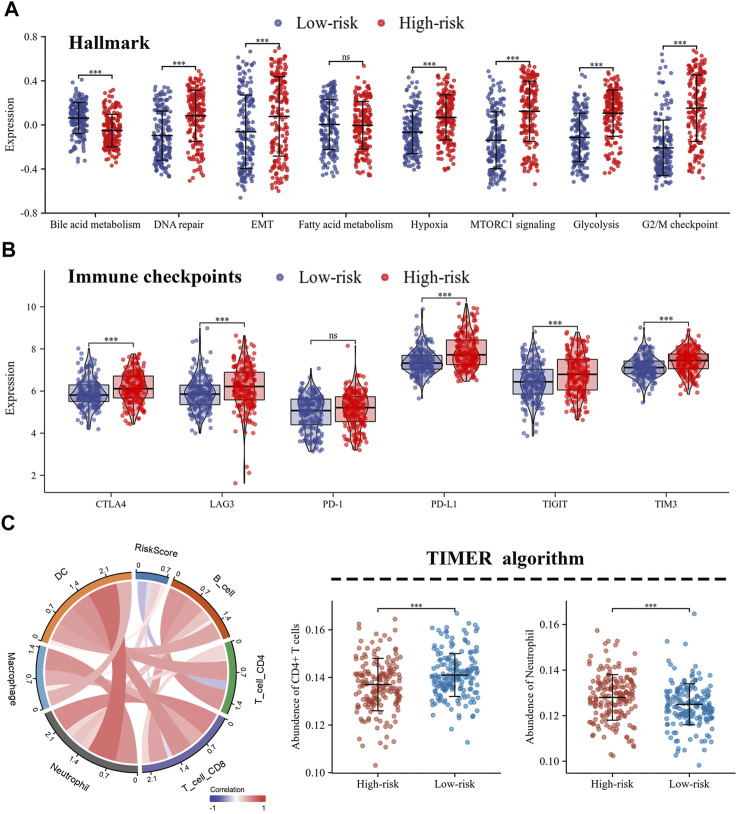
Different characteristics were exhibited between high- and low-risk groups**. (A)** Differences in biological functions between high- and low-risk groups; **(B)** The expression of immune checkpoints in high- and low-risk groups; **(C)** Immune infiltration of 6 immune cell types calculated by TIMER algorithm.

The correlation between risk scores and clinicopathological factors, including gender, age (≤65/>65), smoking history, and pathological stage were evaluated in order. Results indicated that female patients had lower risk scores (Figure 5A), patients with smoking history had higher risk scores (Figure 5C), and patients with pathological stage II had higher risk scores than those with pathological stage I (Figure 5D), yet there was no statistical difference in risk scores by age group (Figure 5B). Indeed, the results of the KM curve were accomplished on different clinicopathological factors. Figures 5E–L showed that the high-risk patients had poor survival time regardless of gender, age, smoking history, and pathological stage. The Hallmark gene sets were downloaded from the MsigDB for Gene Set Variation Analysis (GSVA) to investigate the correlation between risk scores and potential biological functions, and revealed that the low-risk group had higher levels of bile acid metabolism. The high-risk group had higher enrichment levels at the DNA repair, EMT, hypoxia, MTORC1 signaling, glycolysis, and G2/M checkpoint (Figure 6A).

### Distinction of tumor microenvironment based on risk score

**FIGURE 7:**
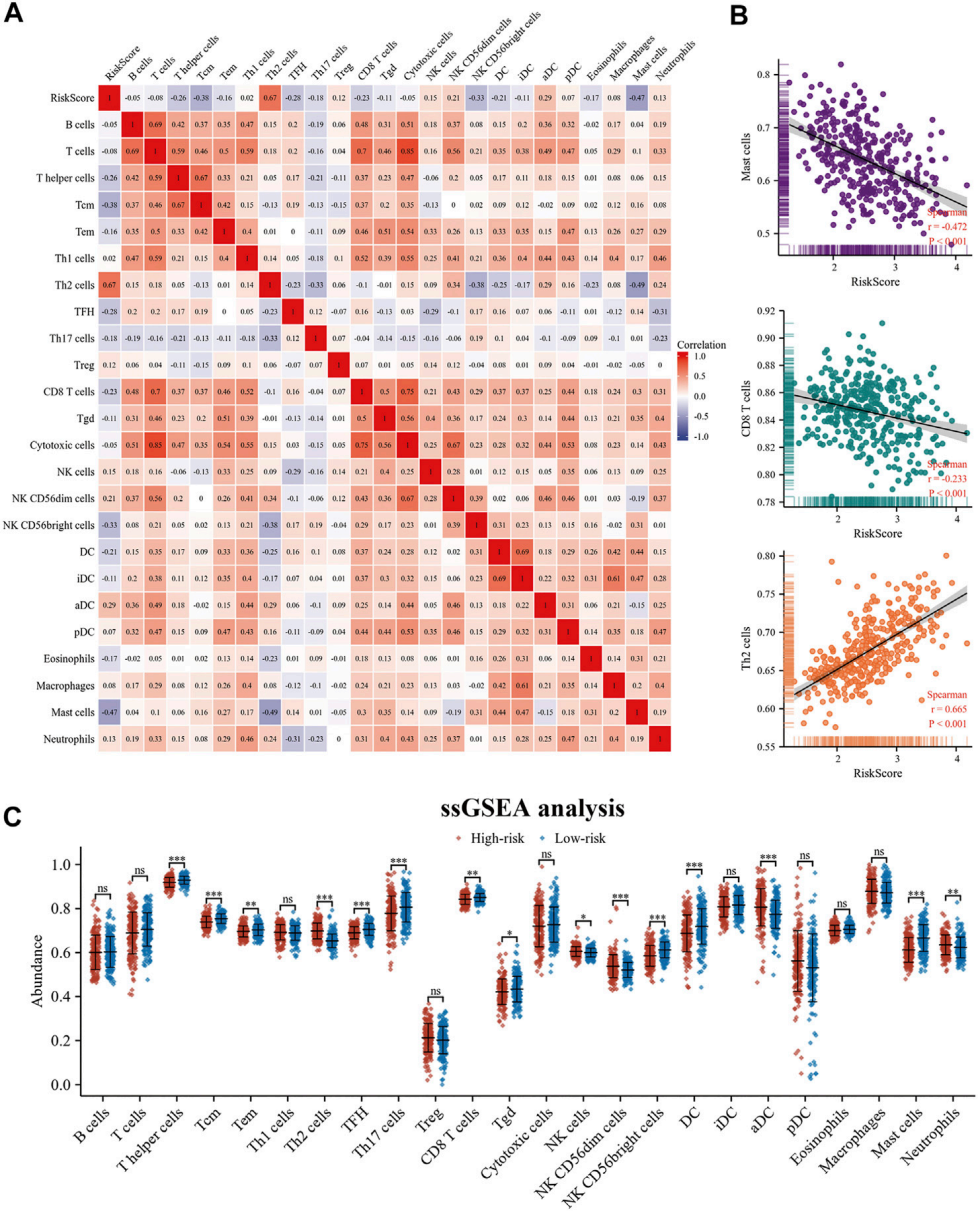
Immune landscape and differences in 24 immune cell types between the high- and low-risk. **(A,B)** The correlation between risk score and 24 immune cell types; **(C)** ssGSEA algorithm analyzed the different abundance of immune cells in the high- and low-risk groups. ssGSEA: single sample Gene Set Enrichment Analysis.

The expression levels of immune co-inhibitory molecules were closely reflected the response to immunotherapy. The comparison of the immune checkpoints between the high- and low-risk groups showed that the high-risk group had higher expression of CTLA4, LAG3, PD-L1, TIGIT and TIM3, while there was no significant difference in the expression of PD-1 (Figure 6B). In addition, the correlation between immune infiltration and risk score was investigated by the TIMER algorithm. The results revealed that the high-risk group had lower abundance of CD4^+^ T cells, while higher abundance of neutrophils (Figures 6C). Further, the ssGSEA algorithm was used to investigate the relationship between the infiltration abundance of 24 immune cell types and the risk score. As shown in Figure 7A,B, risk score was inversely correlated with most cells, including mast cells (r = 0.47) and CD8+T cells (r = 0.23), and was positively correlated with Th2 (r = 0.67). In addition, the comparison of immune cell infiltration abundances between high- and low-risk groups showed the same results. High-risk group had lower abundance of CD8^+^ T cells, DC cells, Mast cells, Tcm cells, TFH cells, while higher abundance of Th2 cells (Figure 7C). These results suggested that tumor progression and tumor escape may occur in patients with a high-risk score.

### Validation of prognostic signatures risk model with GSE72094

**FIGURE 8:**
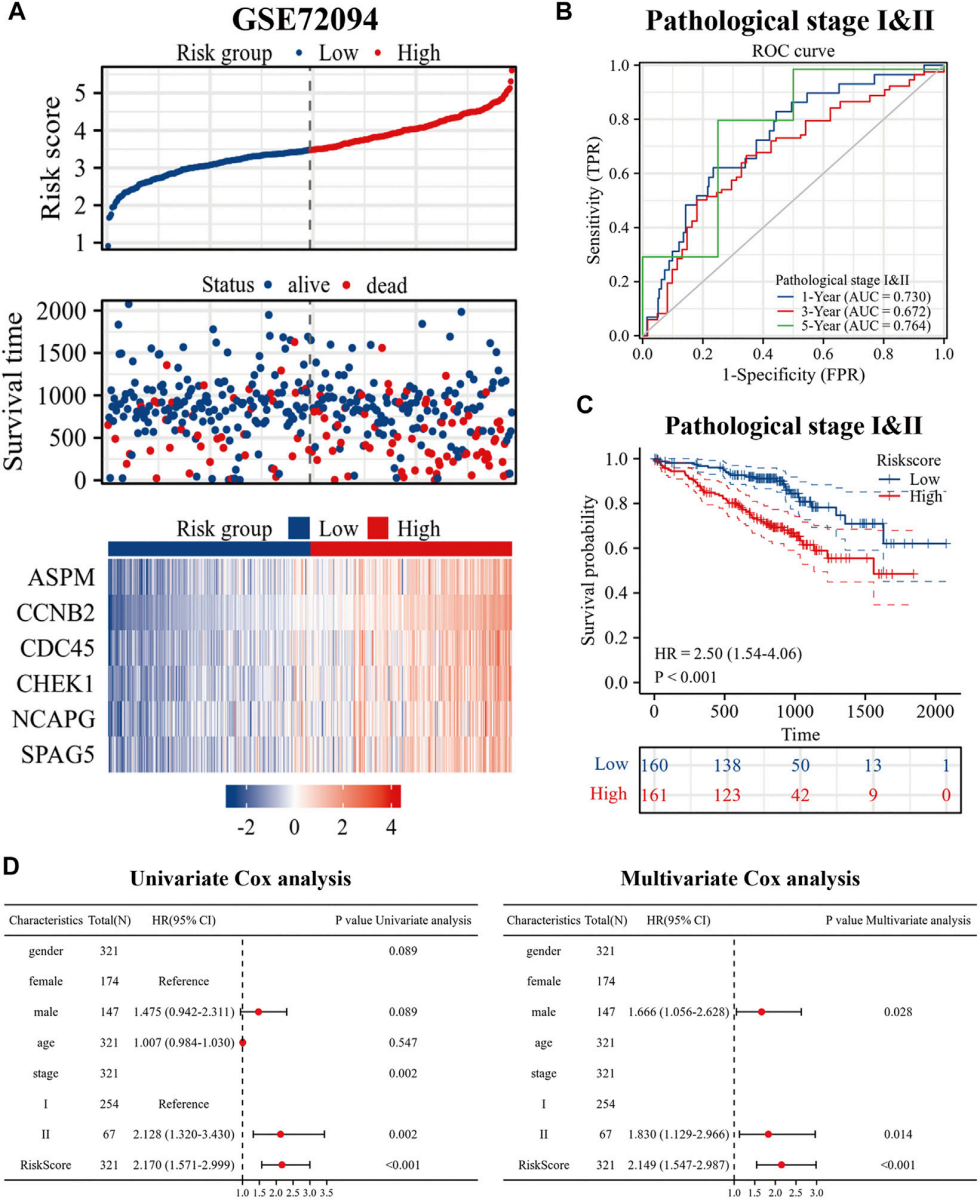
Validation of risk signature. **(A)** Risk factor diagram of es-LUAD patients in GSE72094 dataset; **(B)** ROC curve for GSE72094 dataset; **(C)** KM curve in validation group based on risk score. **(D)** Univariate and Multivariate Cox analysis of risk score and clinical factors. ROC: receiver operating characteristic; KM: Kaplan-Meier.

To better evaluate the prognostic value of our model in patients with es-LUAD, we used an external validation set: GSE72094. Three hundred and twenty-one lung adenocarcinoma patients with pathological stage I and II were processed and extracted. As shown in Figure 8A, there was higher mortality in the es-LUAD patients with a high-risk score, and there were also significant differences in the expression of the six prognostic genes that formed the high accuracy model. Furthermore, we tested the model’s accuracy in predicting survival. The results revealed that the AUC values for 1-year, 3-year, and 5-year overall survival of es-LUAD patients were 0.730, 0.672, and 0.764, respectively (Figure 8B). Es-LUAD patients showed significant prognostic differences at different risk score levels, with worse outcomes in high-risk groups (Figure 8C). Further, Univariate and Multivariate Cox analyses were performed to validate the risk score as an independent prognostic factor (Figure 8D), indicating that the developed model is highly accurate in predicting the es-LUAD prognosis.

### Nomogram based on risk score and clinical factors

**FIGURE 9:**
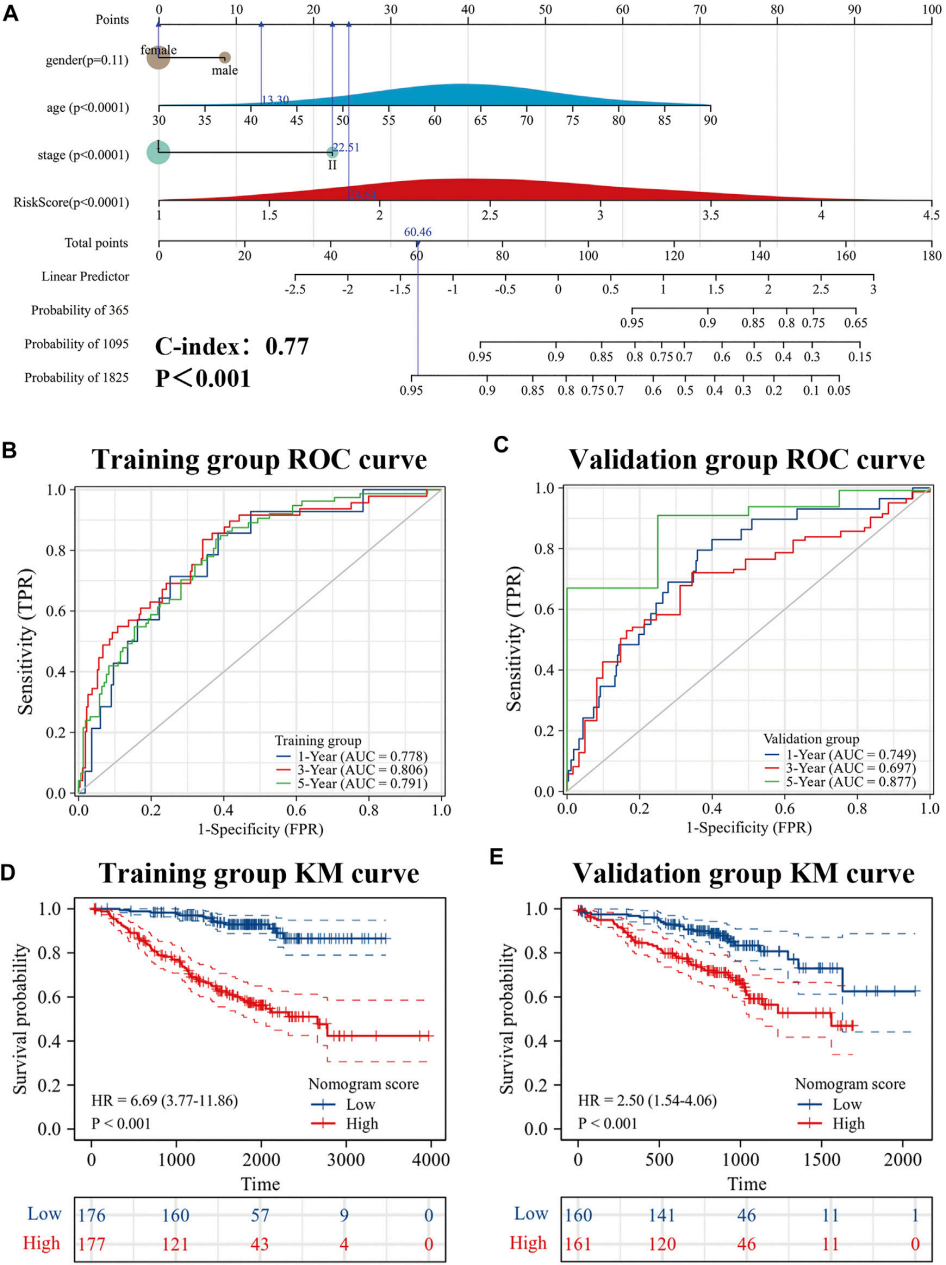
Development of Nomogram. **(A)** Construction of Nomogram by gender, age, pathological stage, and risk score. **(B,C)** 1-, 3- and 5-year ROC curves for training group and validation group; **(D,E)** KM curve for training group and validation group (Based on Nomogram score). KM: Kaplan-Meier.

To further improve the predictive efficacy of the model, gender, age, pathological stage and risk score were combined to construct a nomogram by using the R package “rms.” Prognostic significance was exhibited in 353 samples (The C-index = 0.77 (95% CI: 0.72–0.82), *p*-value < 0.001) (Figure 9A). KM curve analysis showed that the high- and low-levels of nomogram scores could significantly distinguish the prognostic status of es-LUAD patients, and patients with high scores had poor prognoses (Figure 9D). ROC curve analysis showed that the constructed nomogram had higher accuracy in predicting patient survival (Figure 9B). The predicted AUC values for 1-, 3-, and 5-year survival status reached 0.778, 0.806, and 0.791, respectively. To further validate this result, the external data, named the GSE72094, was used to construct and examine the nomogram. The results showed that the model still maintains high accuracy. In the GSE72094 set, patients with es-LUAD with high nomogram scores had a poor prognosis (Figure 9E). Thus, the predicted AUC values of 1-, 3-, and 5-year survival status of patients with es-LUAD significantly reached 0.749, 0.697, and 0.877, respectively (Figure 9C). Taken together, the nomogram model of risk score and clinicopathological characteristics can accuracy the predictive survival in es-LUAD patients.

## Discussion

Copper is a vital “micronutrient” responsible for balancing cell structure and function: excessive copper can hurt various cells and organisms, causing copper-induced cell death. In the presence of unbalanced copper in human serum and tissues, copper promotes tumorigenesis through cancer progression, angiogenesis, and metastasis and acts as a critical cofactor for antioxidant enzymes and multiple forms of cell death (Masuri et al., 2021; Jiang et al., 2022). Previous studies have shown that copper-induced cell death is the mechanism by which inhibition of reactive oxygen species and mitochondrial permeability transition pore affects mitochondrial membrane function (Belyaeva et al., 2012). Another cell death way for inflammatory cells can occur is through cuprizone demyelination and is exhibited in two types: 1) internal death: oxidative stress caused by the alteration of cuprizone contributes to cell body damage of oligodendrocytes; 2) external death: accumulation of immune responses due to disruption of pro-inflammatory cytokines and regulators (Pasquini et al., 2007; Stys et al., 2012; Hooijmans et al., 2019; Zirngibl et al., 2022). Furthermore, the hepatic copper scores were correlated to hepatic neuroinflammatory, apoptosis, malondialdehyde, and fibrosis in various liver diseases (Yamkate et al., 2022), and excessive accumulation of copper can could inhibit mitophagy and promote apoptosis in hepatocytes (Yu et al., 2021). Excitingly, an increasing number of studies have focused on copper-induced cell death. Until recently, Peter Tsvetkov et al. reported a novel type of programmed death: cuproptosis, which has attracted great attention (Tsvetkov et al., 2022). However, no studies have been reported the significance and value of cuproptosis-related genes for guiding clinical treatment of es-LUAD patients. Consequently, it is of great practical importance to classify es-LUAD patients based on cuproptosis-related genes, and to construct a more accurate prognostic model.

LUAD makes up the major histological subgroup of NSCLC. Recently, diagnostic tests for detecting early-stage lung cancer are chest low dose computerized tomography (LDCT) and chest X-rays. However, some es-LUAD patients still have recurrence and metastasis beyond receiving the recommending treatment of the clinical guidelines (Nooreldeen and Bach, 2021; Fu et al., 2022; James et al., 2022). Discovery of new diagnostic tools is essential for evaluating cancer prognosis. With the rapid development of genomics and epigenomics, nearly researches in genomics and epigenomics have paid more attention in the risk stratification, which is also requisite for es-LUAD (Wadowska et al., 2020). In the study, we obtained ten cuproptosis-related genes. Based on expression profile of ten cuproptosis-related genes, the patients were classified into two distinct molecular patterns (cluster1 and cluster2) with notability differences in overall survival (OS) and disease-free survival (DFS): patients with cluster2 had a significantly worse prognosis compared to cluster1. To explore the potential mechanisms underlying the differences in prognosis between the two patterns, we the DEGs of two patterns were identified the DEGs of two patterns and performed functional enrichment analysis of the obtained CR-DEGs. The results of enrichment analysis showed that the CR-DEGs were related to ATPase active molecules, which was consistent with the mechanism that copper induced cell death by TCA cycle proteins (Kahlson and Dixon, 2022; Tsvetkov et al., 2022). Furthermore, we performed the GSEA enrichment analysis showed that cell cycle-related pathways were mostly enriched in cluster2. The results implied that the two different molecular patterns were truly different in the cell cycle function.

To judge the prognosis of individual patients with es-LUAD during the early period, we constructed a cuproptosis pattern-related prognostic model based on hub DEGs that play a central role in two es-LUAD patterns. The prognostic model is an initial exploration of the potential role of cuproptosis pattern-related biomarkers. Briefly, according to the univariate Cox and Lasso-Cox regression analyses, six specific biomarkers were explored in our signature (ASPM, SPAG5, CHEK1, NCAPG, CCNB2, and CDC45). The research has shown that spindle-associated proteins (ASPM and SPAG5), serine/threonine protein kinases (CHEK1), and NCAPG regulated mitotic spindle, segregating chromosome, and coordination of mitotic processes (Ashrafi et al., 2021). ASPM played a substantial role in Wnt signaling which could predict the outcome and survival of pancreatic cancer, as well as promote hepatocellular progression via autophagy (Hsu et al., 2019; Zhang et al., 2021a). Similarly, cyclin family proteins were the key to cancer death signatures. CCNB2 belonged to certain cell cycle control/manufacture proteins and elevates metastatic resistance to synergistic carcinogenesis (Glinsky, 2006). Recent studies have also shown that CDC45 regulated MCM7 in acute myeloid leukemia through the PI3K/AKT pathway, and another mechanism whereby replication partially disrupted eukaryotic DNA replication and triggered by ubiquitination of replication helicases (Zhang et al., 2021b; Jenkyn-Bedford et al., 2021).

In our study, risk score was calculated based on the expression of the six genes, and was considered as an integrated clinical parameter. Depending on the risk score, we could determine the prognosis of es-LUAD patients. In addition, risk score was identified as an independent prognostic factor by univariate Cox and multivariate Cox analyses. The risk score was a powerful complement to pathological stage. It could guide the risk stratification of patients and help clinicians to identify the population with poorer prognosis in es-LUAD early. By combining clinical parameters with the risk score, a more accurate nomogram was constructed. The results of the time-dependent ROC analysis revealed that the predictive efficacy of the nomogram was higher than the risk score. Notably, the predicted AUC values of 1-, 3-, and 5-year survival status reached 0.778, 0.806, and 0.791, respectively, demonstrating a high degree of accuracy. The same results are also obtained in the external independent validation set, which proves the good stability and generalizability of our constructed model.

To further explore the prognostic differences of es-LUAD patients between high- and low-risk groups at the level of molecular mechanisms, we performed GSVA enrichment analysis. We found that high- and low-risk groups showed significant heterogeneity in the enrichment levels of Hallmark gene sets. The high-risk group had higher enrichment levels for functional features such as DNA repair, EMT, hypoxia, MTORC1 signaling, glycolysis, and G2/M checkpoint, while the low-risk group had higher enrichment levels for Bile acid metabolism. This result explained the high activation of tumor progression and metastasis in patients with high-risk scores. The TME, where tumor cells live, plays a pivotal role in tumorigenesis, development and metastasis. Research has shown that the TME of LUAD patients is highly heterogeneous, and this difference in TME may also indirectly contribute to the poor survival status of some es-LUAD patients (Hinshaw and Shevde, 2019). In our study, we performed the immune infiltration analysis for patients in the high- and low-risk groups separately to look for differences in immune cell abundance. Excitingly, the high-risk score of es-LUAD had lower abundance of CD8^+^ T cells, DC cells, Tcm cells, and TFH cells. CD8+T cells, as key immune cells in tumor immunity, can specifically recognize and kill tumor cells (Raskov et al., 2021). In addition, numerous studies have also shown that DC cells, Tcm cells, and TFH cells are involved in fighting tumor progression (Liu et al., 2020; Wculek et al., 2020; Cui et al., 2021). Therefore, patients in the high-risk group exhibit an immunosuppressed TME that contributes more to tumor immune escape.

Due to the highly effective therapeutic effects, immune checkpoint inhibitors (ICIs) have made great progress in the application of cancer treatment, yet studies have found that only a fraction of patients are sensitive to immunotherapy (Zhang and Zhang, 2020). Therefore, an in-depth exploration of the model we constructed has potential value to guide the strategy of ICIs usage. In this study, we analyzed the expression of immune co-inhibitory molecules in the high- and low-risk groups separately. We found that the high-risk group had higher expression levels of CTLA4, LAG3, PD-L1, TIGIT, and TIM3. Previous studies have shown that the expression of immune co-inhibitory molecules correlates with the level of tumor escape and the effect of immunotherapy, so this result offers a new idea to identify es-LUAD with higher malignant risk and provide targeted adjuvant treatment strategies. In conclusion, unlike other prognostic models that include advanced lung adenocarcinoma, we focused on es-LUAD patients, an easily overlooked population (Fane and Weeraratna, 2020; Stoletov et al., 2020). By constructing a cuproptosis pattern-related prognostic model with high accuracy, we uncovered the potential clinical application of the risk score, which is expected to help clinicians in the future.

Nowadays, numerous cell death-related prognostic models have been published, such as apoptosis-related gene model (Zou et al., 2022), ferroptosis-related gene model (Zhong et al., 2022), and autophagy-related gene model (Deng et al., 2022). Unlike them, we for the first time confirm that the expression of cuproptosis pattern-related genes correlates with es-LUAD patients prognosis, and have a high predictive efficacy. However, there are certain shortcomings in our study. Our study is mainly based on secondary analysis of public databases, and therefore further experimental validation for the mechanism of cuproptosis in cancer is needed.

## Conclusion

Cuproptosis-related genes may contribute to the classification of es-LUAD. The model constructed by cuproptosis pattern-related genes allowed risk stratification of es-LUAD patients and revealed differences in tumor microenvironment between different risk groups. In addition, the Nomogram based on risk score and clinical factors can accurately predict the survival status of es-LUAD patients, and may serve as an essential reference to guide clinical decision-making.

## Data Availability

Publicly available datasets were analyzed in this study. This data can be found here: The data supporting the conclusions was downloaded from GEO database (https://www.ncbi.nlm.nih.gov/geo/). Sangerbox platform and Xiantao Academic platform provide support for code calculation and visualization of results. In addition, the R code used in the article can be obtained from the corresponding author upon reasonable request.

## Abbreviations

Es-LUAD, Early-stage lung adenocarcinoma; NSCLC, Non-small cell lung cancer; CR-DEGs, Cuproptosis-related differentially expressed genes; PCD, Programmed cell death; KM, Kaplan Meier; HR, hazard ratio; GSEA, Gene set enrichment analysis; TCA, Mitochondrial tricarboxylic acid; GO, Gene ontology; KEGG, Kyoto encyclopedia of genes and genome; GSEA, Gene set enrichment analysis; DLAT, Dihydrolipoamide S-acetyl transferase; PPI, Protein-protein Interaction; ssGSEA, single sample Gene set enrichment analysis; NS, No significance.

## References

[B1] Alexander M. Kim S. Y. Cheng H. (2020). Update 2020: Management of non-small cell lung cancer. Lung 198 (6), 897–907. 10.1007/s00408-020-00407-5 33175991 PMC7656891

[B2] Anusewicz D. Orzechowska M. Bednarek A. K. (2020). Lung squamous cell carcinoma and lung adenocarcinoma differential gene expression regulation through pathways of Notch, Hedgehog, Wnt, and ErbB signalling. Sci. Rep. 10 (1), 21128. 10.1038/s41598-020-77284-8 33273537 PMC7713208

[B3] Ashrafi F. Ghezeldasht S. A. Ghobadi M. Z. (2021). Identification of joint gene players implicated in the pathogenesis of HTLV-1 and BLV through a comprehensive system biology analysis. Microb. Pathog. 160, 105153. 10.1016/j.micpath.2021.105153 34419613

[B4] Belyaeva E. A. Sokolova T. V. Emelyanova L. V. Zakharova I. O. (2012). Mitochondrial electron transport chain in heavy metal-induced neurotoxicity: Effects of cadmium, mercury, and copper. ScientificWorldJournal. 2012, 136063. 10.1100/2012/136063 22619586 PMC3349094

[B5] Bergmann A. (2007). Autophagy and cell death: No longer at odds. Cell 131 (6), 1032–1034. 10.1016/j.cell.2007.11.027 18083090 PMC2502067

[B6] Cui C. Wang J. Fagerberg E. Chen P. M. Connolly K. A. Damo M. (2021). Neoantigen-driven B cell and CD4 T follicular helper cell collaboration promotes anti-tumor CD8 T cell responses. Cell 184 (25), 6101–6118 e13. 10.1016/j.cell.2021.11.007 34852236 PMC8671355

[B7] Deng J. Zhang Q. Lv L. Ma P. Zhang Y. Zhao N. (2022). Identification of an autophagy-related gene signature for predicting prognosis and immune activity in pancreatic adenocarcinoma. Sci. Rep. 12 (1), 7006. 10.1038/s41598-022-11050-w 35488119 PMC9054801

[B8] Dixon S. J. Lemberg K. M. Lamprecht M. R. Skouta R. Zaitsev E. M. Gleason C. E. (2012). Ferroptosis: An iron-dependent form of nonapoptotic cell death. Cell 149 (5), 1060–1072. 10.1016/j.cell.2012.03.042 22632970 PMC3367386

[B9] Fane M. Weeraratna A. T. (2020). How the ageing microenvironment influences tumour progression. Nat. Rev. Cancer 20 (2), 89–106. 10.1038/s41568-019-0222-9 31836838 PMC7377404

[B10] Fu R. Zhang J. T. Chen R. R. Li H. Tai Z. X. Lin H. X. (2022). Identification of heritable rare variants associated with early-stage lung adenocarcinoma risk. Transl. Lung Cancer Res. 11 (4), 509–522. 10.21037/tlcr-21-789 35529798 PMC9073742

[B11] Garon E. B. Hellmann M. D. Rizvi N. A. Carcereny E. Leighl N. B. Ahn M. J. (2019). Five-year overall survival for patients with advanced non‒small-cell lung cancer treated with pembrolizumab: Results from the phase I KEYNOTE-001 study. J. Clin. Oncol. 37 (28), 2518–2527. 10.1200/JCO.19.00934 31154919 PMC6768611

[B12] Glinsky G. V. (2006). Genomic models of metastatic cancer: Functional analysis of death-from-cancer signature genes reveals aneuploid, anoikis-resistant, metastasis-enabling phenotype with altered cell cycle control and activated polycomb group (PcG) protein chromatin silencing pathway. Cell Cycle 5 (11), 1208–1216. 10.4161/cc.5.11.2796 16760651

[B13] Hanzelmann S. Castelo R. Guinney J. (2013). Gsva: Gene set variation analysis for microarray and RNA-seq data. BMC Bioinforma. 14, 7. 10.1186/1471-2105-14-7 PMC3618321 23323831

[B14] Hinshaw D. C. Shevde L. A. (2019). The tumor microenvironment innately modulates cancer progression. Cancer Res. 79 (18), 4557–4566. 10.1158/0008-5472.CAN-18-3962 31350295 PMC6744958

[B15] Hooijmans C. R. Hlavica M. Schuler F. A. F. Good N. Good A. Baumgartner L. (2019). Remyelination promoting therapies in multiple sclerosis animal models: A systematic review and meta-analysis. Sci. Rep. 9 (1), 822. 10.1038/s41598-018-35734-4 30696832 PMC6351564

[B16] Hsu C. C. Liao W. Y. Chan T. S. Chen W. Y. Lee C. T. Shan Y. S. (2019). The differential distributions of ASPM isoforms and their roles in Wnt signaling, cell cycle progression, and pancreatic cancer prognosis. J. Pathol. 249 (4), 498–508. 10.1002/path.5341 31465125 PMC6899738

[B17] James A. N. Conroy L. R. Hawkinson T. R. Chang J. E. Manauis E. C. Lamb J. F. (2022). High-fat/high-carbohydrate diet increases glycogen accumulation in lung tissue *in vivo* . FASEB J. 36. 1. 10.1096/fasebj.2022.36.s1.r2115

[B18] Jenkyn-Bedford M. Jones M. L. Baris Y. Labib K. P. M. Cannone G. Yeeles J. T. P. (2021). A conserved mechanism for regulating replisome disassembly in eukaryotes. Nature 600 (7890), 743–747. 10.1038/s41586-021-04145-3 34700328 PMC8695382

[B19] Jiang Y. Huo Z. Qi X. Zuo T. Wu Z. (2022). Copper-induced tumor cell death mechanisms and antitumor theragnostic applications of copper complexes. Nanomedicine (Lond) 17 (5), 303–324. 10.2217/nnm-2021-0374 35060391

[B20] Johnson W. E. Li C. Rabinovic A. (2007). Adjusting batch effects in microarray expression data using empirical Bayes methods. Biostatistics 8 (1), 118–127. 10.1093/biostatistics/kxj037 16632515

[B21] Kahlson M. A. Dixon S. J. (2022). Copper-induced cell death. Science 375 (6586), 1231–1232. 10.1126/science.abo3959 35298241

[B22] Kanehisa M. Furumichi M. Sato Y. Ishiguro-Watanabe M. Tanabe M. (2021). Kegg: Integrating viruses and cellular organisms. Nucleic Acids Res. 49 (D1), D545–D551. 10.1093/nar/gkaa970 33125081 PMC7779016

[B23] Kerr J. F. Wyllie A. H. Currie A. R. (1972). Apoptosis: A basic biological phenomenon with wide-ranging implications in tissue kinetics. Br. J. Cancer 26 (4), 239–257. 10.1038/bjc.1972.33 4561027 PMC2008650

[B24] Linehan A. Forde P. M. (2020). Moving immunotherapy into early-stage lung cancer. Cancer J. 26 (6), 543–547. 10.1097/PPO.0000000000000493 33298726

[B25] Liu Q. Sun Z. Chen L. (2020). Memory T cells: Strategies for optimizing tumor immunotherapy. Protein Cell 11 (8), 549–564. 10.1007/s13238-020-00707-9 32221812 PMC7381543

[B26] Masuri S. Vaňhara P. Cabiddu M. G. Moran L. Havel J. Cadoni E. (2021). Copper(II) phenanthroline-based complexes as potential AntiCancer drugs: A walkthrough on the mechanisms of action. Molecules 27 (1), 49. 10.3390/molecules27010049 35011273 PMC8746828

[B27] Mazzone P. J. Silvestri G. A. Souter L. H. Caverly T. J. Kanne J. P. Katki H. A. (2021). Screening for lung cancer: CHEST guideline and expert panel report. Chest 160 (5), e427–e494. 10.1016/j.chest.2021.06.063 34270968 PMC8727886

[B28] National Lung Screening Trial Research T. Church T. R. Black W. C. Aberle D. R. Berg C. D. Clingan K. L. (2013). Results of initial low-dose computed tomographic screening for lung cancer. N. Engl. J. Med. 368 (21), 1980–1991. 10.1056/NEJMoa1209120 23697514 PMC3762603

[B29] Nooreldeen R. Bach H. (2021). Current and future development in lung cancer diagnosis. Int. J. Mol. Sci. 22 (16), 8661. 10.3390/ijms22168661 34445366 PMC8395394

[B30] Pasquini L. A. Calatayud C. A. Bertone Una A. L. Millet V. Pasquini J. M. Soto E. F. (2007). The neurotoxic effect of cuprizone on oligodendrocytes depends on the presence of pro-inflammatory cytokines secreted by microglia. Neurochem. Res. 32 (2), 279–292. 10.1007/s11064-006-9165-0 17063394

[B31] Raskov H. Orhan A. Christensen J. P. Gogenur I. (2021). Cytotoxic CD8(+) T cells in cancer and cancer immunotherapy. Br. J. Cancer 124 (2), 359–367. 10.1038/s41416-020-01048-4 32929195 PMC7853123

[B32] Ritchie M. E. Phipson B. Wu D. Hu Y. Law C. W. Shi W. (2015). Limma powers differential expression analyses for RNA-sequencing and microarray studies. Nucleic Acids Res. 43 (7), e47. 10.1093/nar/gkv007 25605792 PMC4402510

[B33] Shi J. Zhao Y. Wang K. Shi X. Wang Y. Huang H. (2015). Cleavage of GSDMD by inflammatory caspases determines pyroptotic cell death. Nature 526 (7575), 660–665. 10.1038/nature15514 26375003

[B34] Siegel R. L. Miller K. D. Fuchs H. E. Jemal A. (2022). Cancer statistics, 2022. Ca. Cancer J. Clin. 72 (1), 7–33. 10.3322/caac.21708 35020204

[B35] Stoletov K. Beatty P. H. Lewis J. D. (2020). Novel therapeutic targets for cancer metastasis. Expert Rev. Anticancer Ther. 20 (2), 97–109. 10.1080/14737140.2020.1718496 31997674

[B36] Stys P. K. Zamponi G. W. van Minnen J. Geurts J. J. G. (2012). Will the real multiple sclerosis please stand up? Nat. Rev. Neurosci. 13 (7), 507–514. 10.1038/nrn3275 22714021

[B37] Subramanian A. Tamayo P. Mootha V. K. Mukherjee S. Ebert B. L. Gillette M. A. (2005). Gene set enrichment analysis: A knowledge-based approach for interpreting genome-wide expression profiles. Proc. Natl. Acad. Sci. U. S. A. 102 (43), 15545–15550. 10.1073/pnas.0506580102 16199517 PMC1239896

[B38] Sun L. Wang H. Wang Z. He S. Chen S. Liao D. (2012). Mixed lineage kinase domain-like protein mediates necrosis signaling downstream of RIP3 kinase. Cell 148 (1-2), 213–227. 10.1016/j.cell.2011.11.031 22265413

[B39] Sung H. Ferlay J. Siegel R. L. Laversanne M. Soerjomataram I. Jemal A. (2021). Global cancer statistics 2020: GLOBOCAN estimates of incidence and mortality worldwide for 36 cancers in 185 countries. Ca. Cancer J. Clin. 71 (3), 209–249. 10.3322/caac.21660 33538338

[B40] Taminau J. Meganck S. Lazar C. Steenhoff D. Coletta A. Molter C. (2012). Unlocking the potential of publicly available microarray data using inSilicoDb and inSilicoMerging R/Bioconductor packages. BMC Bioinforma. 13, 335. 10.1186/1471-2105-13-335 PMC3568420 23259851

[B41] Tibshirani R. (1997). The lasso method for variable selection in the Cox model. Stat. Med. 16 (4), 385–395. 10.1002/(sici)1097-0258(19970228)16:4<385::aid-sim380>3.0.co;2-3 9044528

[B42] Tsvetkov P. Coy S. Petrova B. Dreishpoon M. Verma A. Abdusamad M. (2022). Copper induces cell death by targeting lipoylated TCA cycle proteins. Science 375 (6586), 1254–1261. 10.1126/science.abf0529 35298263 PMC9273333

[B43] Vanlangenakker N. Vanden Berghe T. Vandenabeele P. (2012). Many stimuli pull the necrotic trigger, an overview. Cell Death Differ. 19 (1), 75–86. 10.1038/cdd.2011.164 22075985 PMC3252835

[B44] Wadowska K. Bil-Lula I. Trembecki L. Sliwinska-Mosson M. (2020). Genetic markers in lung cancer diagnosis: A review. Int. J. Mol. Sci. 21 (13), E4569. 10.3390/ijms21134569 32604993 PMC7369725

[B45] Wang H. Lengerich B. J. Aragam B. Xing E. P. (2019). Precision lasso: Accounting for correlations and linear dependencies in high-dimensional genomic data. Bioinformatics 35 (7), 1181–1187. 10.1093/bioinformatics/bty750 30184048 PMC6449749

[B46] Wculek S. K. Cueto F. J. Mujal A. M. Melero I. Krummel M. F. Sancho D. (2020). Dendritic cells in cancer immunology and immunotherapy. Nat. Rev. Immunol. 20 (1), 7–24. 10.1038/s41577-019-0210-z 31467405

[B47] Wilkerson M. D. Hayes D. N. (2010). ConsensusClusterPlus: A class discovery tool with confidence assessments and item tracking. Bioinformatics 26 (12), 1572–1573. 10.1093/bioinformatics/btq170 20427518 PMC2881355

[B48] Yamkate P. Lidbury J. A. Steiner J. M. Suchodolski J. S. Giaretta P. R. (2022). Immunohistochemical expression of oxidative stress and apoptosis markers in archived liver specimens from dogs with chronic hepatitis. J. Comp. Pathol. 193, 25–36. 10.1016/j.jcpa.2022.02.005 35487620

[B49] Yu W. Liao J. Yang F. Zhang H. Chang X. Yang Y. (2021). Chronic tribasic copper chloride exposure induces rat liver damage by disrupting the mitophagy and apoptosis pathways. Ecotoxicol. Environ. Saf. 212, 111968. 10.1016/j.ecoenv.2021.111968 33550083

[B50] Zhang Y. Zhang Z. (2020). The history and advances in cancer immunotherapy: Understanding the characteristics of tumor-infiltrating immune cells and their therapeutic implications. Cell. Mol. Immunol. 17 (8), 807–821. 10.1038/s41423-020-0488-6 32612154 PMC7395159

[B51] Zhang H. Yang X. Zhu L. Li Z. Zuo P. Wang P. (2021). ASPM promotes hepatocellular carcinoma progression by activating Wnt/β-catenin signaling through antagonizing autophagy-mediated Dvl2 degradation. FEBS Open Bio 11 (10), 2784–2799. 10.1002/2211-5463.13278 PMC8487047 34428354

[B52] Zhang R. Liu Z. Zhang G. (2021). CDC45 modulates MCM7 expression and inhibits cell proliferation by suppressing the PI3K/AKT pathway in acute myeloid leukemia. Am. J. Transl. Res. 13 (9), 10218–10232. 34650692 PMC8507005

[B53] Zhong H. Wang Y. Jia J. Yang H. Zhang H. Li T. (2022). Ferroptosis related genes are regulated by methylation and predict the prognosis of glioblastoma patients. Transl. Cancer Res. 11 (4), 603–614. 10.21037/tcr-21-2470 35571655 PMC9091034

[B54] Zirngibl M. Assinck P. Sizov A. Caprariello A. V. Plemel J. R. (2022). Oligodendrocyte death and myelin loss in the cuprizone model: An updated overview of the intrinsic and extrinsic causes of cuprizone demyelination. Mol. Neurodegener. 17 (1), 34. 10.1186/s13024-022-00538-8 35526004 PMC9077942

[B55] Zou X. He R. Zhang Z. Yan Y. (2022). Apoptosis-related signature predicts prognosis and immune microenvironment infiltration in lung adenocarcinoma. Front. Genet. 13, 818403. 10.3389/fgene.2022.818403 35571020 PMC9094710

