# Supplementary material for: Cuproptosis-related gene signature correlates with the tumor immune features and predicts the prognosis of early-stage lung adenocarcinoma patients
Source: Front Genet. 2022 Sep 14;13:977156. doi: 10.3389/fgene.2022.977156 (PMC9515444; doi:10.3389/fgene.2022.977156)
Supplement: Supplementary file 3 [file Table1.DOCX]

**Table S1:** The clinic character of LUAD patients from GEO databases.

|  | **Training group** | | **Validation group** |
| --- | --- | --- | --- |
|  | GSE31210 | GSE50081 | GSE72094 |
|  |  |  |  |
| **Status** |  |  |  |
| Live | 191 | 76 | 244 |
| Dead | 35 | 51 | 77 |
| **Survival Time (Year)** |  |  |  |
| ＜1 | 4 | 16 | 48 |
| ≥1,＜3 | 37 | 27 | 212 |
| ≥3,＜5 | 83 | 30 | 57 |
| ≥5 | 102 | 54 | 4 |
| **Gender** |  |  |  |
| male | 105 | 65 | 147 |
| female | 121 | 62 | 174 |
| **Stage** |  |  |  |
| I | 168 | 92 | 254 |
| II | 58 | 35 | 67 |
| **Smoking** |  |  |  |
| never | 115 | 23 | 27 |
| ever | 111 | 92 | 244 |
| unknow | 0 | 12 | 50 |
| **Age(Year)** |  |  |  |
| ＜65 | 164 | 40 | 86 |
| ≥65 | 62 | 87 | 235 |
