# Supplementary figures and images for: Cuproptosis-related gene signature correlates with the tumor immune features and predicts the prognosis of early-stage lung adenocarcinoma patients
Source: Front Genet. 2022 Sep 14;13:977156. doi: 10.3389/fgene.2022.977156 (PMC9515444; doi:10.3389/fgene.2022.977156)

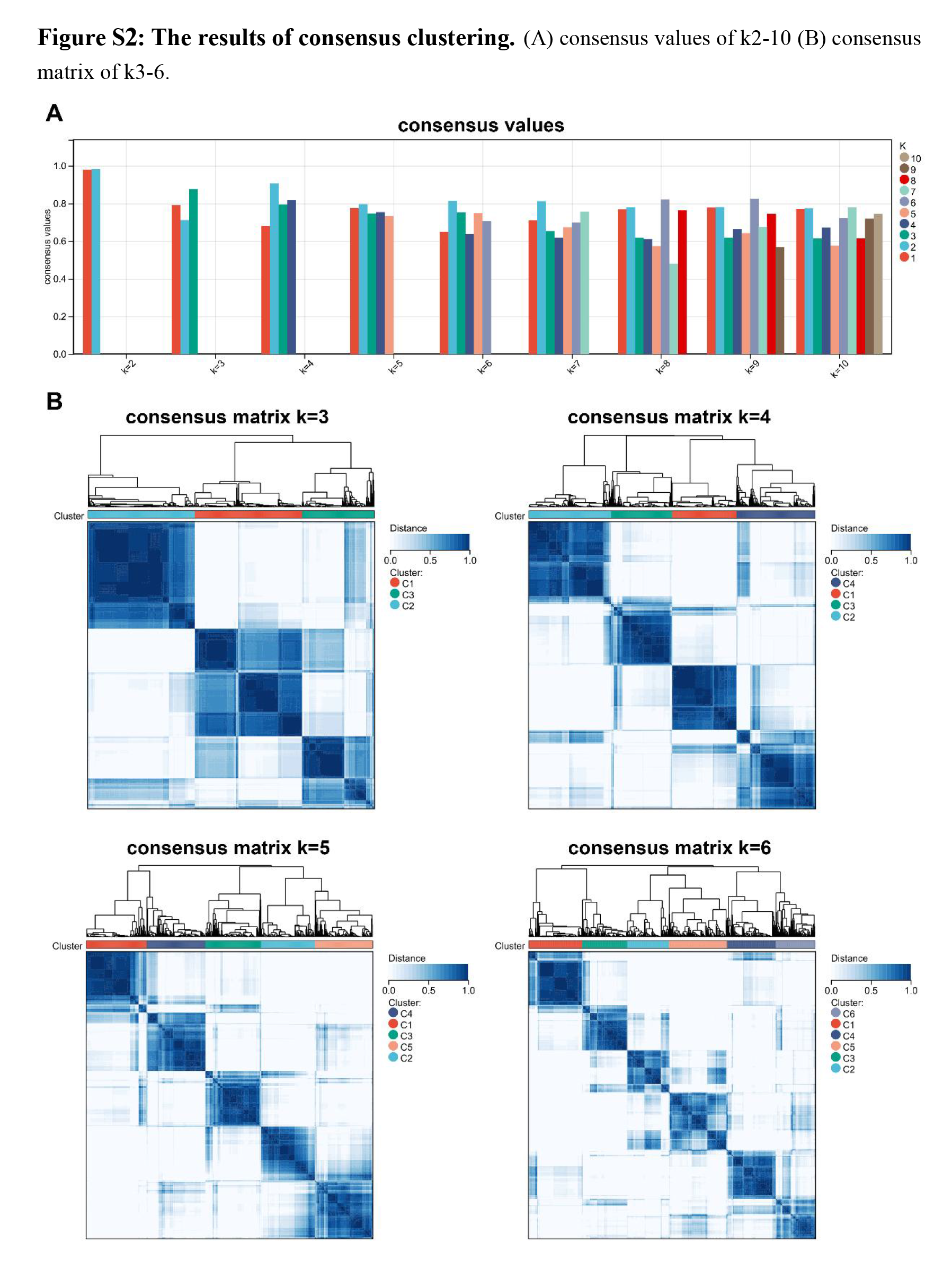

Supplement: Supplementary file 4 [file Image2.TIF]

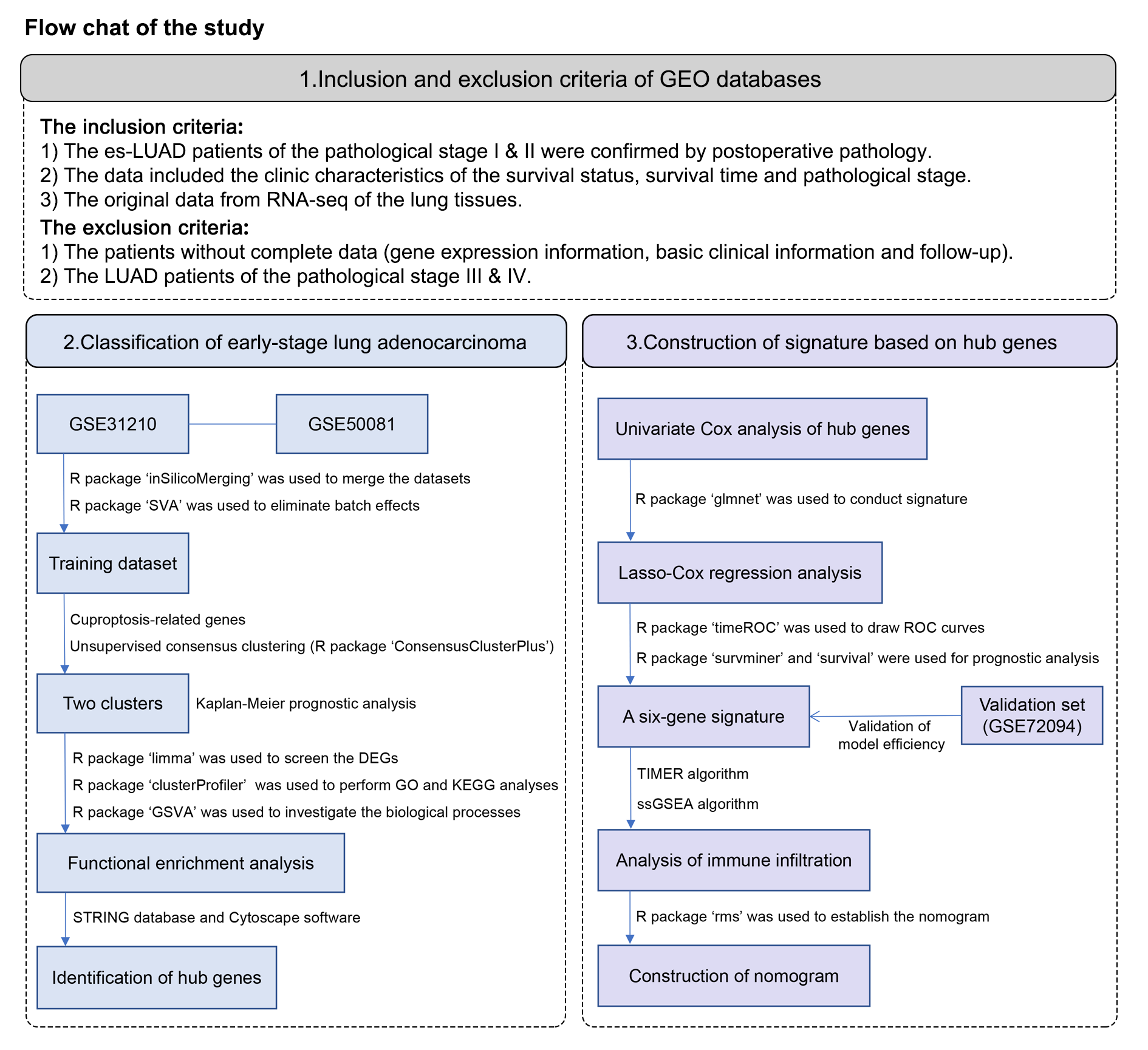

Supplement: Supplementary file 5 [file Image1.TIF]
